# More on hepatic granulomas

**DOI:** 10.1186/s13000-015-0442-6

**Published:** 2015-11-19

**Authors:** Resat Ozaras, Mucahit Yemisen, Ilker Inanc Balkan

**Affiliations:** Infectious Diseases Department, Cerrahpasa Medical School, Istanbul University, TR-34098 Cerrahpasa, Istanbul Turkey

**Keywords:** Hepatic granuloma, Surgical staples, Chronic hepatitis

## Abstract

We have read the case report of Nihon-Yanagi et al. The patient they described developed hepatic granuloma two times and the granulomatous lesion was surrounding metal staples/clips suggesting that the granuloma was due to surgical staples/clips.

Hepatic granulomas (HGs) are reported in around 5 % of patient who undergo a liver biopsy and caused by several diseases including sarcoidosis, tuberculosis, hydatid cyst, brucellosis, typhoid fever, chronic hepatitis B and C and primary biliary cirrhosis (PBC). Chronic hepatitis B and C infections are the most common and serious causes of liver damage in patient with renal failure. Their prevalence is a higher than people without renal failure. We have previously reported that the prevalences of HGs in patients with chronic hepatitis B and C are 1.5 and 1.3 % respectively. The described patient was on hemodialysis for 12 years. The other causes of HG seem excluded; however hepatitis B and C infections and PBC should have been tested and excluded before ascribing the HGs to surgical staples/clipping material.

## Letters to the Editor

Dear Sir,

We have read the case report of Nihon-Yanagi et al. [1] with great interest. The patient they described developed hepatic granuloma(HG) two times and the granulomatous lesion was surrounding metal staples/clips suggesting that the granuloma was due to surgical/staples/clips.

HGs are reported in around 5 % of patient who undergo a liver biopsy and caused by several diseases including sarcoidosis, tuberculosis, hydatid cyst, brucellosis, typhoid fever, chronic hepatitis B and C, and primary biliary cirrhosis (PBC) [2, 3]. Chronic hepatitis B and C infections are the most common and serious causes of liver damage in patients with renal failure [4]. Their prevalence is a higher than people without renal failure. We have previously reported that the prevalences of HGs in patients with chronic hepatitis B and C are 1.5 and 1.3 % respectively [5, 6]. The described patient was on hemodialysis for 12 years.

Another cause of HBs is PBC. It is an immune-mediated cholestatic liver disease characterized by destruction of cholangiocytes. The histology includes a portal tract inflammatory infiltrate composed of plasma cells, mononuclear cells, and neutrophils [7]. Noncaseating epithelioid granulomas are seen especially in early-stage disease. The diagnosis is made by the presence of the antimitochondrial antibody which is found in 95 % of patients.

In chronic liver disease and PBC, the described granulomas are microscopic granulomas and the ones in the presented case are macroscopic granulomas. However it is not known whether the causes of microscopic granulomas may also cause macroscopic granulomas or contribute to the development of macroscopic granulomas of any other causes.

The other causes of HG seem excluded; however hepatitis B and C infections and PBC should have been tested and excluded before ascribing the HGs to surgical staples/clipping material.

## Abbreviations

HG: Hepatic granulomas
PBC: Primary biliary cirrhosis
